# A Review of Vulvovaginal Atrophy as Part of Genitourinary Syndrome of Menopause

**DOI:** 10.7759/cureus.113672

**Published:** 2026-07-30

**Authors:** Corrie DeGraffenreid, Emmanuel Amabebe

**Affiliations:** 1 Obstetrics and Gynecology, The University of Texas Medical Branch, Galveston, USA

**Keywords:** genetics, genitourinary syndrome of menopause, menopause, vaginal estrogen, vulvovaginal atrophy

## Abstract

In this narrative review, we evaluate and discuss current literature on the assessment of vulvovaginal atrophy as part of the genitourinary syndrome of menopause. Publications discussing clinical evaluation methods, including physical exam, cytology, pH, and symptom questionnaires, as well as laboratory evaluation through histology and genetic analysis, were reviewed. A wide variety of methods are used to clinically evaluate the signs and symptoms of atrophy; however, these tools lack consistency and validation across publications. We also appraised the pertinent publications evaluating the genetic changes in the vagina occurring with menopause and subsequent treatment. More research is needed to validate and standardize clinical evaluation techniques and to further define the genetic pathway alterations to develop targeted therapies in the future.

## Introduction and background

Vulvovaginal atrophy, a component of the genitourinary syndrome of menopause (GSM), affects about 40% of postmenopausal individuals [1,2]. The term "GSM" is used to encompass the genitourinary signs and symptoms of menopause [3]. Estrogen depletion alters vaginal vascularity, collagen, musculature, epithelium, and microbiome, leading to menopausal changes including increased dryness, epithelial fragility, and pH, and decreased elasticity and cellular maturation [1,4,5]. Unlike vasomotor symptoms, genitourinary signs and symptoms progress with aging without treatment [3].

Methods to evaluate GSM include pelvic examination, vaginal pH, cytology, and questionnaires [6]. Laboratory analysis of the vaginal wall is limited, and few studies have examined vulvovaginal atrophy histologic changes [2,7]. Recent advances have evaluated genetic and metabolic changes of atrophy; however, the mechanisms driving these are not fully understood. Herein, we examine current methods to assess vulvovaginal atrophy and highlight new perspectives for future research on GSM.

## Review

Methods

A broad literature search was conducted using Ovid and PubMed databases. Terms were used to search a broad range of vaginal atrophy components, including ‘vaginal atrophy’, ‘menopause’, ‘physical exam’, ‘laboratory test’, ‘histology’, ‘questionnaire’, ‘estrogen therapy’, and ‘gene expression’. Publication date was limited to 1990 or later to allow for a more in-depth search in the setting of limited publications on the topic. Identified resources were uploaded into Mendeley Reference Manager software to store and analyze the literature search results. Both original articles and review articles were included. Studies that were not available in English translation were excluded.

Clinical and laboratory evaluation

Pelvic Examination

Pelvic examination is part of the initial vulvovaginal atrophy clinical evaluation. Exam findings include pallor, friability, petechiae or bleeding, dryness, decreased epithelial rugae, vaginal length and diameter, fissures or ulcerations, and mucosal thinning [2,3]. Various scoring systems published largely lack standardization or validation [2].

The vaginal physical examination scale is a validated tool assessing vaginal friability, petechiae, conization, and rugae [8]. Other scoring indices include the global atrophy score, urogenital atrophy criteria, vaginal atrophy index, and vaginal health index, among others [9-12]. These assess a range of atrophy components with various severity rating systems. The intra- and interobserver validity and reproducibility of these scoring indices have not been reported.

Symptom Evaluation

Vulvovaginal atrophy and GSM symptoms do not always correlate with physical findings and should be evaluated regardless of exam findings [2,3]. A wide range of symptoms may be present, ranging from vaginal dryness, burning, irritation, and soreness to urinary symptoms including dysuria, urgency, frequency, and recurrent urinary tract infections to sexual symptoms including dyspareunia, bleeding, and soreness [2,3].

Several questionnaires have been studied to evaluate GSM symptoms. The Urogenital Atrophy Questionnaire is a 45-item tool encompassing a wide range of symptoms and is validated in breast cancer survivors regardless of sexual orientation or activity [2,13]. The Vulvovaginal Symptom Questionnaire is a 21-item survey modeled on the Skindex-16 used for skin diseases and is validated in multiple populations, including English-, Spanish-, Turkish-, and Brazilian Portuguese-speaking groups [6,14-16]. The FDA proposed the Most Bothersome Symptom Survey to identify and rate the most common and severe symptoms, tracking them through treatment to monitor improvement [17]. Other questionnaires published lack validation or assess a broader range of menopause symptoms, with few questions focusing on GSM and vulvovaginal atrophy [2].

Vaginal pH

Decreased estrogen in menopause leads to a basic shift of the vaginal pH [2,18,19]. Pre-menopause, the estrogenized vagina promotes glycogen accumulation that is converted by lactobacilli into lactic acid to maintain pH between 3.5 and 4.5 [20]. As estrogen declines and the vaginal epithelium thins, glycogen production and lactobacilli decrease, while anaerobic, gut, and skin species predominate [4,21-23]. Previous studies suggest a pH above 5 indicates decreased serum estradiol, rising follicle-stimulating hormone, and menopause status [24,25]. Common factors can skew pH results, including pH increase due to blood, cervical mucus, infection, semen, and medications, and pH decrease with smoking [24,26].

Testing methods reported include pH paper or indicator strips, nitrazine paper, and pH probes with various ranges of pH measured [2,18]. Measuring the pH of the lateral vaginal wall is validated and correlated with vaginal cytology, with pH above 5.0 indicating increased parabasal cell presence [7,18,27-30]. The FDA-recommended minimum pH of 5.0 for inclusion in vulvovaginal atrophy studies is used in several publications [17,29,31-33]. Other studies advocate for a cutoff of 4.5 in the absence of vaginitis, and the literature indicates similar sensitivity to serum follicle-stimulating hormone levels of at least 40 IU/L, which is used for menopause diagnosis [18,24,25,34,35]. The tools and methods used to test pH and pH cutoff values are not standardized [2].

Cytology and Vaginal Maturation Index

Pre-menopause, estrogen stimulates vaginal epithelial cell proliferation, maturation, and glycogen accumulation [23,36]. As estrogen declines, epithelial cells fail to mature due to the inability to produce intracellular glycogen, leaving predominantly immature parabasal and intermediate cells and few mature superficial cells [37]. The vaginal maturation index (VMI) quantifies the degree of vaginal cell maturity and indicates atrophy severity. The VMI specimen is prepared from exfoliated vaginal squamous cells [38]. It is reported and read systematically as % parabasal/ % intermediate/ % superficial cells, and the VMI value is calculated using one of the numerous formulas reported [2]. One frequently used formula is calculated as (0 × % parabasal cells) + (0.5 × % intermediate cells) + (1.0 × % superficial cells) [2,39]. A VMI left shift, corresponding to a lower VMI value, indicates immature parabasal cell predominance and mature superficial cell absence, representing a hypoestrogenic state. A VMI right shift with a higher VMI value indicates superficial epithelial cell predominance and higher estrogen levels [39].

VMI and the percent of superficial cells present are used for research eligibility criteria, but standard cutoff values are lacking [2,40-42]. Studies using vaginal cell maturity frequently use a VMI cutoff of near 50 or less, or <5% superficial cells present [40-42]. Other factors influencing VMI variation include patient and interpreter factors and slide preparation methods. Patient factors include smoking and body mass index (BMI) [43-45]. Higher BMI is associated with increased circulating estrogen, which may increase VMI scores, while smoking is associated with lower VMI, possibly related to increased androgens in smokers [20,45-48]. Improper specimen collection influences results. Lastly, intra- and interobserver slide interpretation variation and lack of trained personnel can generate VMI result inconsistencies.

Outcomes are mixed on correlation between VMI and atrophy signs and symptoms, with lower VMI observed in patients with significant atrophy on examination [7,49-51]. Tracking VMI in patients receiving estrogen can assess therapy response, with a lower VMI observed in atrophic patients receiving placebo compared to those receiving estrogen [52-54].

**Figure 1:**
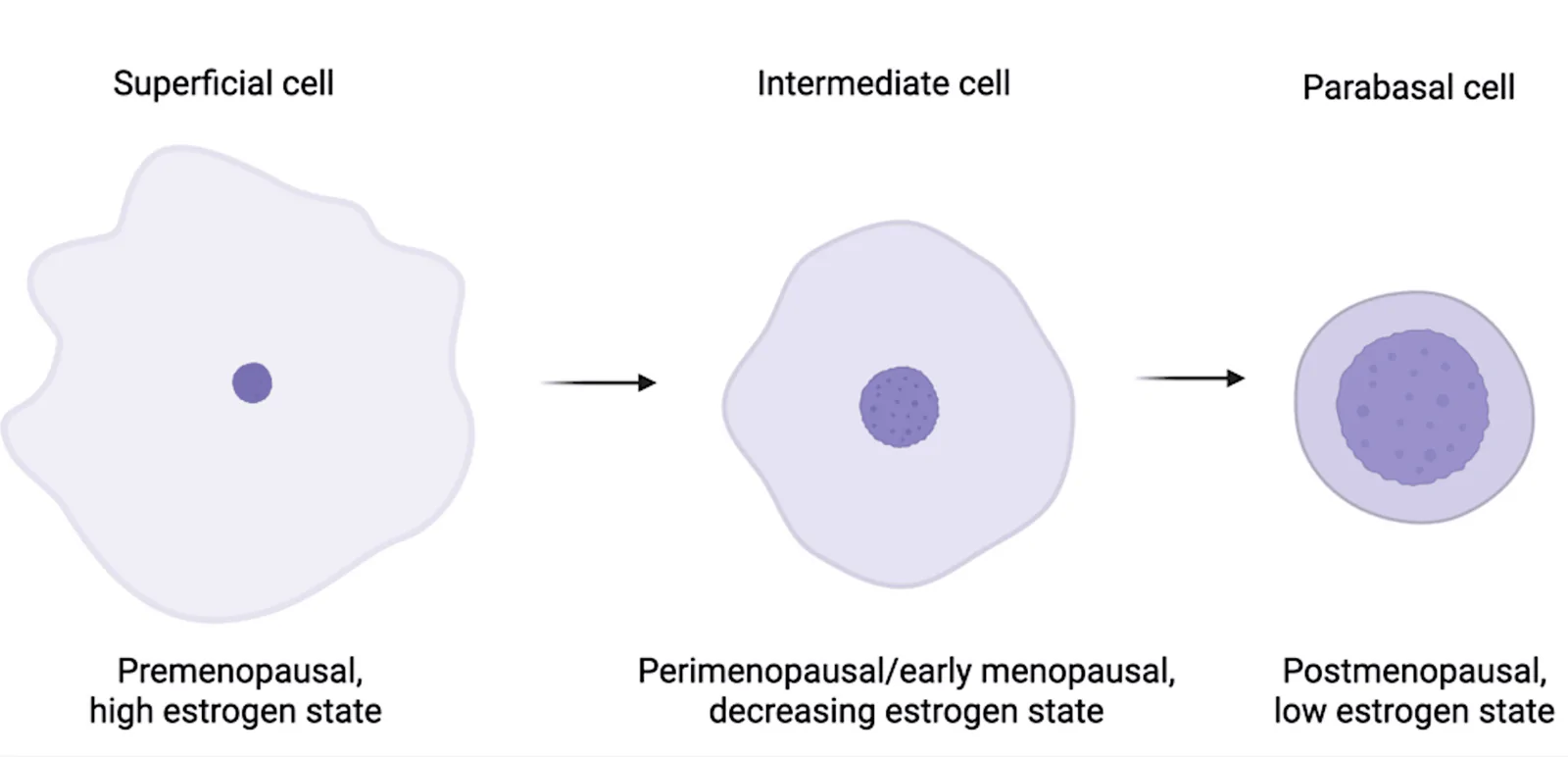
Vaginal epithelial cell depiction from the premenopausal, high estrogen state to the postmenopausal, low estrogen state Image Credit: Corrie DeGraffenreid Created in BioRender.com. DeGraffenreid, C. (2026) https://BioRender.com/8ylew8a

Cytomorphology of vaginal cells has also been assessed, consistent with VMI findings [27,55]. In atrophic vaginas not receiving estrogen, the nuclear circumference and area are significantly greater than in estrogen-treated tissues (Figure 1), consistent with parabasal cell predominance in atrophic tissue versus superficial cells in premenopausal or estrogen-treated tissue.

Histology

**Figure 2:**
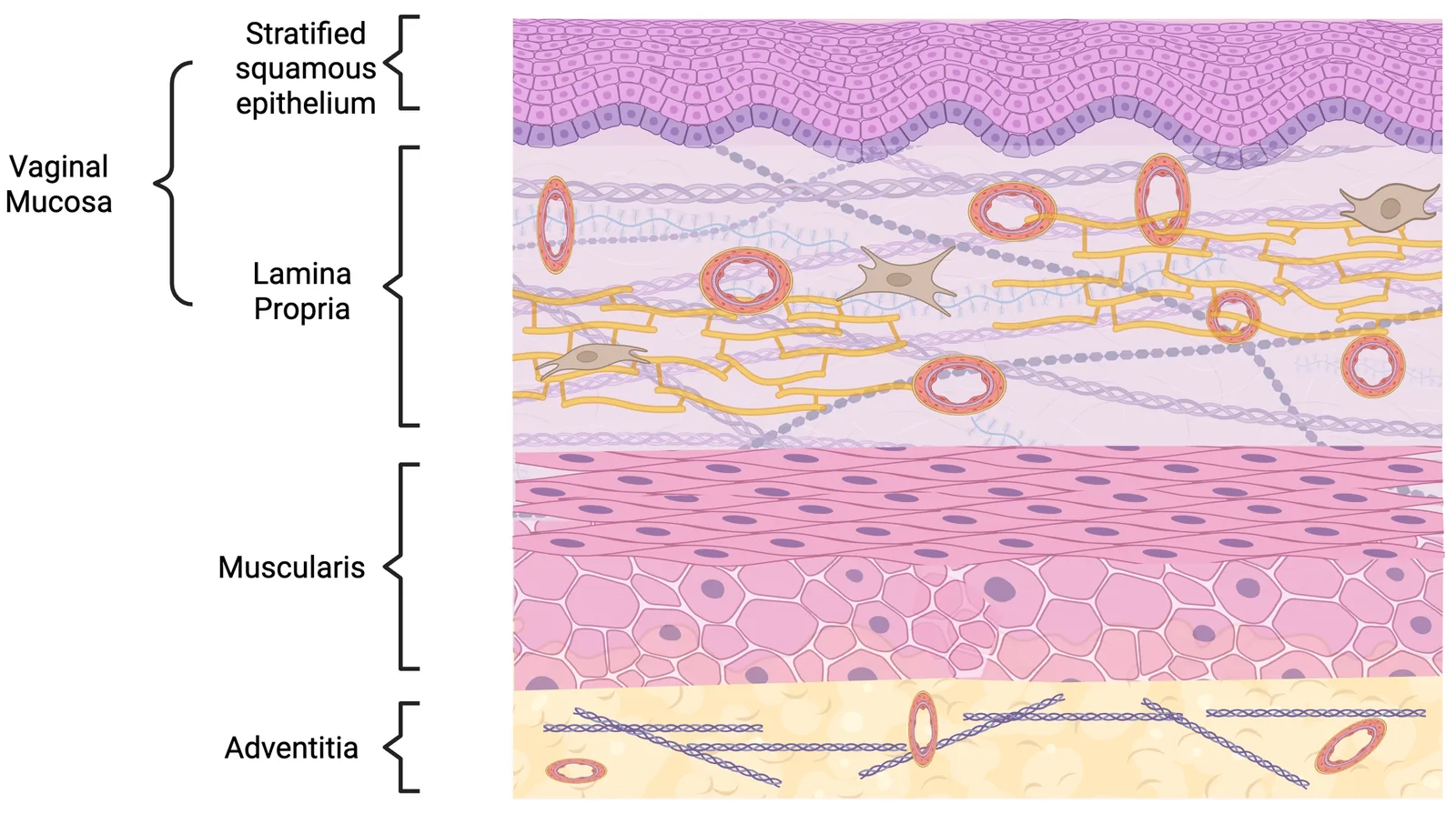
Layers of the vaginal wall including vasculature and collagen, elastin, and fibroblast components Image Credit: Corrie DeGraffenreid Created in BioRender.com. DeGraffenreid, C. (2026) https://BioRender.com/zxu8t8f

Vaginal wall histology provides insight into vulvovaginal atrophy changes. The vaginal wall is composed of four layers and fluctuates in thickness throughout an individual’s lifetime [56]. The non-keratinized stratified squamous epithelium and lamina propria, a vascularized, glandless connective tissue layer comprised of elastin, collagen, and fibroblasts, form the vaginal mucosa. The underlying fibromuscular layer, the muscularis, supports the vaginal mucosa with smooth muscle, collagen, and elastin. The deepest layer, the adventitia, is loose connective tissue composed of collagen, elastin, and adipose with supporting vasculature, lymphatics, and nerves (Figure 2) [56,57].

The estrogen-responsive vaginal lining progressively thins in menopause [58]. The mucosal thickness decreases as age increases, with average thickness decreasing from 1.6 mm in individuals 35 years or younger to 1.1 mm after age 55 [57]. The epithelial and muscularis layers appear most affected by estrogen, while the lamina propria maintains similar thickness between pre- and post-menopause and between postmenopausal patients using estrogen versus those not [57,59]. Superficial epithelial cell loss contributes to thinning epithelium and low VMI [40,50,55,60]. With estrogen treatment, epithelial thickness nearly doubles, as noted by pre- and post-estrogen treatment measurements of depth and epithelial cell counts [61,62]. Estrogen may increase fibromuscular thickness by increasing collagen levels. This is not seen in vaginal mucosa, where collagen content does not increase in postmenopausal individuals using estrogen [59]. Rete-ridge undulation, the epithelial projections of the dermal-epithelial interface, are significantly decreased in atrophy [60]. Rete ridges provide epithelial structural support and secure it to underlying connective tissue, and loss of this stability decreases structural integrity while worsening tissue sensitivity in menopause [60].

Pathophysiologic changes

Maintenance of premenopausal vaginal integrity and development of menopausal changes involve a complex network of hormonal, immunologic, metabolic, and other cell signaling pathways that are altered by a hypoestrogenic state [1,19,60,63,64]. Research has begun to evaluate these pathways, including the gene expression alterations occurring in vaginal tissue when estrogen is decreased or blocked, and how estrogen replacement can restore physiological functions.

Metabolic

Decreased estrogen causes downregulation of critical vaginal homeostasis processes and upregulation of processes causing the pathologic changes of menopause (Table 1). There is a significant decrease in genetic processes involved in epithelial cell differentiation and metabolism and an increase in processes related to blood vessel morphogenesis, cell migration, tissue remodeling, cellular cytoskeleton organization, wound response, and immune response [65].

**Table 1:** Vulvovaginal atrophy genetic alterations associated with metabolic processes ↑ indicates protein is upregulated; ↓ indicates protein is downregulated; w/ tx: with estrogen treatment

Gene	Protein	Alteration	Function
PLAT	Plasminogen activator, tissue type	↑	Fibrinolysis, inflammatory response
VEGF	Vascular endothelial growth factor	↑	Promotes angiogenesis
COL1A1	Collagen type I alpha 1 chain	↓	Pro-alpha1 chain of type I collagen, a fibril-forming collagen in connective tissues
COL1A2	Collagen type I alpha 2 chain	↓	Pro-alpha2 chain of type I collagen
COL3A1	Collagen type III alpha 1 chain	↓	Pro-alpha1 chain of type III collagen, a fibril-forming collagen in connective tissues
				COL5A2	Collagen type V alpha 2 chain	↓	Alpha chain of low abundance fibrillar collagen type V; regulates fiber assembly of type I and type V collagens
LOX	Lysyl oxidase	↓	Crosslinking of collagen and elastin				
PLOD3	Procollagen-lysine,2-oxoglutarate 5-dioxygenase 3	↓	Role in stability of collagen intermolecular crosslinks
LUM	Lumican	↓	regulate collagen fibril organization, epithelial cell migration, and tissue repair
DPT	Dermatopontin	↓	Involved in fibril formation and extracellular matrix assembly and interactions
				MFAP4	Microfibril associated protein 4	↑	Extracellular matrix elastic fiber organization
ALOX12	Arachidonate 12-lipoxygenase	↓	Epidermal barrier formation in cell differentiation
RPTN	Repetin	↓	component of cornified envelope; involved in epithelial differentiation
LCE3E	Late cornified envelope 3E	↓	Epithelial differentiation and keratinization
LCE3D	Late cornified envelope 3D	↓	Epithelial differentiation and keratinization
GJB2	Gap junction protein beta 2	↓	Epithelial differentiation and gap junction functions
DSG1	Desmoglein 1	↑ w/tx	Desmosome component; structural integrity of vaginal epithelium function
KRT1	Keratin 1	↑ w/tx	Type II cytokeratin; forms keratinocyte cytoskeleton
KRT2A	Keratin 2	↑ w/tx	Type II keratinocyte cytokeratin; role in structural integrity and epidermal mechanical resilience
KRT6B	Keratin 6B	↑ w/tx	Type II cytokeratin forming intermediate filaments; function in epithelial structural integrity, mechanical stress resilience, and cell signaling and migration
KRT10	Keratin 10	↑ w/tx	Type I cytokeratin forming intermediate filaments of epithelial cell cytoskeleton
KRT16	Keratin 16	↑ w/tx	Type I cytokeratin forming intermediate filaments of epithelial cell cytoskeleton
KRTDAP	Keratinocyte differentiation associated protein	↑ w/tx	Regulates keratinocyte differentiation and maintenance of stratified epithelium
TGM1	Transglutaminase 1	↑ w/tx	Role in forming cornified envelope of epidermis
TGM3	Transglutaminase 3	↑ w/tx	Role in forming cornified envelope of epidermis
SPRR1-3	Small Proline-Rich Protein 1, 2, and 3	↓	Cornified envelope protein; function in keratinocyte differentiation; epithelial differentiation marker

Vascular and coagulation pathway alterations are seen in atrophy. PLAT gene product, a fibrinolytic enzyme that breaks down thrombus plasminogen and functions in tissue injury inflammatory response, is increased in atrophy [66]. Increased PLAT product may contribute to increased bleeding, inflammation, and tissue sensitivity seen with atrophy [67]. VEGF signaling pathway upregulation is observed with postmenopausal estrogen use, indicating attempts to re-establish vaginal vascularity [65].

Tissue structure pathways are altered in postmenopausal vaginal tissue, significantly impacting the pelvic floor connective tissue [68]. Collagen, the predominant connective tissue protein, is downregulated. There is a significant decrease in major structural collagens (COL1A1, COL1A2, COL3A1) and low-abundance collagens (COL5A2) [60]. Collagen upregulation, especially collagen type I, is seen with estrogen therapy. Collagen type I is a ligamentous tissue organized fiber component while collagen type III is found in loose areolar tissue. The former has favored upregulation localized to the muscularis with estrogen treatment [59]. Estrogen use alters matrix metalloproteinase (e.g., MMP9, MMP12) activity, suggesting suppression of collagenase breakdown of collagen with treatment [59]. Lysyl oxidase (LOX), which alters collagen and elastin for collagen fibril organization, is downregulated in atrophy, while genes facilitating fibril formation, including PLOD3, LUM, and DPT, are upregulated [60]. MFAP4, encoding an extracellular matrix glycoprotein with elastic fiber organization function, is also upregulated. Alterations to structural genes suggest menopausal elastic fibril formation dysregulation, which could cause changes to tissue structure and integrity seen with atrophy [69].

Other atrophy-altered genes in the literature involve epithelial cell development, structure, differentiation, and repair. Epithelial differentiation genes are downregulated, including ALOX12, RPTN, LCE3E, LCE3D, and GJB2 [60,70]. TP63, a transcription factor regulating epithelial cell morphogenesis, is decreased [71]. TP63 directly induces ALOX12, which promotes epidermal barrier formation, further supporting decreased ALOX12 expression [72]. ALOX12 expression is correlated with declining estrogen levels and worsened vulvovaginal atrophy scores on exam [70]. ALOX12 is downregulated in patients taking aromatase inhibitors that block androgen conversion to estrogen [70]. Immunohistochemistry protein staining shows ALOX12 localized to the basal epithelium, with decreased staining intensity in postmenopausal patients using aromatase inhibitors compared to estrogen therapy. Epithelial development and repair genes, including LCE3D, DSG1, KRTDAP, KRT2A, ALOX12, and ALOX12B, are upregulated in postmenopausal individuals on estrogen compared to non-users [70].

Epithelial cell development pathways involving genes of keratin synthesis, keratinocyte differentiation, and keratinization are impacted. In healthy vaginal mucosa, the basal and suprabasal layers undergoing terminal differentiation express cytokeratin. Unlike epidermal epithelium, they do not form distinct keratin bundles [73]. Keratins are markers of epithelial cell differentiation in patients receiving estrogen, which induces upregulation of keratin genes, including KRT1, KRT2A, KRT6B, KRT10, KRT16, TGM1, TGM3, SPRR1, SPRR2, SPRR3, LCE3D, DSG1, and KRTDAP. Decreased expression of these genes compromises tissue proliferation and barrier function of atrophic vaginal epithelium [65]. Genes involved in cornified envelope formation and maintenance are downregulated in individuals with severe dryness reported on questionnaires and seen on exams [67,74]. Without the cornified envelope epithelial barrier function, epithelial cells become sensitive to drying, microbial infection, and chemical agents.

SPRR1-3 are downregulated in atrophy and upregulated with estrogen treatment, indicating atrophy affects epithelial cell differentiation [75]. This estrogen-induced expression improves physical barrier function, supported by the estrogen-induced upregulation of DSG1, an epithelial cell junction protein that maintains tissue integrity and strength [65].

Immune Response

Atrophic vagina has increased immune response, consistent with the low-grade inflammation observed in aging tissue (Table 2) [76]. Complement genes important for apoptotic cell clearance and linked to aging are upregulated [77]. C3, CFB, and CFH show increased expression, and CFH is increased in postmenopausal patients with vaginal dryness [67]. KRT17 protein, an intermediate filament in wounded epithelium, is upregulated in inflammatory conditions and significantly expressed in postmenopausal vagina [78,79]. Matrix metalloproteinases and CXCL chemokines are upregulated in patients with vaginal dryness [67]. MMP7 upregulation, which opposes SPINK proteases that prevent extracellular matrix degradation, leads to increased inflammation and breakdown of the extracellular matrix [67,80]. Upregulated CXCL chemokines recruit monocytes and leukocytes to the vaginal epithelium. With estrogen therapy, CXCL6 and MMP10 are reduced, leading to decreased inflammation and increased vascular integrity [65]. Innate and adaptive immune mediator downregulation of IL-8, IL-19, CXCL1, CXCL6, and WFDC2 is also observed, indicating estrogen decreases inflammation, improves barrier function, lowers immune cell recruitment, and reduces pathogen exposure [65,81]. Estrogen may upregulate other immune response components (IL-1F5, CRISP2, CRISP3, and CDA) that may protectively adapt to vaginal microbiota changes and improve pathogen defense. Immune cell apoptosis may affect immune response downregulation with estrogen use, exhibited by decreased leukocyte-expressed chemokines and cytokines and increased PTEN and apoptotic pathways [65].

**Table 2:** Vulvovaginal atrophy genetic alterations associated with immune response ↑ indicates protein is upregulated; ↓ indicates protein is downregulated; w/ tx: with estrogen treatment

Gene	Protein	Alteration	Function
C3	Complement component 3	↑	Complement component involved in activating the complement system; modulates inflammation; antimicrobial activity
CFB	Complement factor B	↑	Alternative complement activation pathway component; localized to the major histocompatibility complex class III gene cluster involved in immune reaction
CFH	Complement factor H	↑	Component of the Regulator of Complement Activation gene cluster involved in regulating complement activation for innate defense against microbial infections
KRT17	Keratin 17	↑	Type I intermediate filament expressed in epidermal tissues
MMP7	Matrix metallopeptidase 7	↑	Involved in extracellular matrix degradation
MMP10	Matrix metallopeptidase 10	↓ w/ tx	Involved in breakdown of extracellular matrix, anti-inflammatory and tissue healing role
				CXCL1	C-X-C motif chemokine ligand 1	↓ w/ tx	Immune and inflammatory response, antimicrobial action
CXCL6	C-X-C motif chemokine ligand 6	↓ w/ tx	Immune and inflammatory response, antibacterial action				
				WFDC2	WAP four-disulfide core domain 2	↓ w/ tx	Role in innate immunity
IL-8	Interleukin 8	↓ w/ tx	Major mediator of inflammatory response
IL-19	Interleukin 19	↓ w/ tx	Major mediator of inflammatory response
IL-1F5/IL-36Ra	Interleukin 36 receptor antagonist	↑ w/tx	Inhibits the inflammatory effects of other IL-1 family members
CRISP2	Cysteine-rich secretory protein 2	↑ w/tx	Involved in cell-cell adhesion and immune response
				CRISP3	Cysteine-rich secretory protein 3	↑ w/tx	Involved in innate immunity and inflammation
CDA	Cytidine deaminase	↑ w/tx	Essential to DNA and RNA synthesis; involved in immune response, including host defense

Hormone Receptors

Estrogen, through interaction with its receptors, functions in epithelial cell differentiation, maturation, and maintenance of the vaginal barrier [60,65,73,82]. Estrogen receptor alpha (ERα) functions in cell proliferation, while estrogen receptor beta (ERβ) is involved in cellular differentiation [83]. Both receptors have decreased expression in postmenopausal vaginal tissue (Table 3) [83,84]. While ERα expression is more mildly decreased, ERβ expression is markedly decreased, even with estrogen treatment [85-87]. This is seen in both the anterior and posterior vagina. As estrogen levels and estrogen receptor expression decline, the elastic and lubricative vaginal functions decrease due to vascularity loss, disturbance of supportive collagen and elastin, and impaired cellular proliferation and differentiation, leading to the signs and symptoms seen in atrophy [46,88,89]. With topical estrogen, receptor expression increases; however, ERβ expression seems less treatment responsive, and neither receptor reaches pre-menopausal levels with therapy despite its reported effect on atrophy objectively and subjectively [46,83,86-88,90]. ERα is an upstream regulator of several genes when comparing postmenopausal patients on aromatase inhibitors versus those using vaginal estrogen, but its expression is not significantly altered between these groups [70]. The progesterone receptor is regulated by 17-beta estradiol and shows decreased expression in atrophied tissue, consistent with decreased menopausal estrogen levels [60,91]. With estrogen therapy, the progesterone receptor increases expression in vaginal tissue [83].

**Table 3:** Hormone receptor related vulvovaginal atrophy genetic alterations ↑ indicates protein is upregulated; ↓ indicates protein is downregulated

Gene	Protein	Alteration	Function
ERα	Estrogen receptor 1	↓	Nuclear receptor that is activated by estrogen; encoded by the ESR1 gene
ERβ	Estrogen receptor beta	↓	Nuclear receptor that is activated by estrogen; encoded by the ESR2 gene
PGR	Progesterone receptor	↓	Nuclear receptor that is activated by progesterone
GREB1	Growth regulating estrogen receptor binding 1	↓	Estrogen-responsive gene functions in regulating proliferation of estrogen-responsive tissues
JUP	Junction plakoglobin	↓	Protein component of desmosomes and intermediate junctions involved in cell-to-cell adhesion and suppresses epithelial cell proliferation
DSC2	Desmocollin 2	↓	desmocollins involved in cell-cell junctions that resist shearing forces; expression within basal/suprabasal keratinocytes in squamous epithelium
PCDH8	Protocadherin 8	↑	involved in cell adhesion, proliferation, differentiation, and signaling

Genes interacting with estrogen receptors have decreased expression in atrophy compared to increased expression with estrogen therapy. GREB1, an estrogen-induced gene that regulates proliferation of estrogen-responsive tissues, is downregulated in atrophy [60,92,93]. Menopause alters genes indirectly interacting with ERα, including downregulated JUP and DSC2 and upregulated PCDH8 [70].

JUP suppresses epithelial proliferation and aids cell-to-cell adhesion [94]. DSG1 and DSC2 work with JUP to maintain desmosome function and prevent JUP degradation to preserve its expression [70,95]. Decreased JUP is seen with anti-estrogen treatment; however, immunohistochemical staining indicates increased JUP protein intensity in the basal epithelium in these patients, secondary to sequestration to prevent destruction [94,96]. The increased JUP presence in vaginal epithelium could be a critical component of the low vaginal cell proliferation in estrogen-depleted patients. Downregulation of JUP, DSC2, and DSG in these patients could be due to the thinned vaginal mucosa, where these proteins normally function [70]. Lastly, PCDH8 is involved in cell adhesion, proliferation, differentiation, and signaling [97,98]. It plays a tumor suppressive role in some cancer types, preventing cellular proliferation and promoting apoptosis [99,100]. Menopausal PCDH8 upregulation and its indirect ERα interaction could inhibit vaginal mucosa proliferation in atrophy if it acts in a suppressive manner, as seen in certain cancers.

## Conclusions

In this review, we presented an overview of the current literature on vulvovaginal atrophy as part of GSM. We described the current methods of clinical evaluation and the evolving data on gene-regulating pathways that lead to vulvovaginal atrophy. Although we have presented a comprehensive review of available literature, a systematic review could provide a more robust critical appraisal of publications. However, a systematic review would be premature due to the lack of methodological consistency and the paucity of existing studies on atrophy and GSM.

Despite multiple methods to assess and diagnose atrophy, a standard approach is not established. Further research is needed to standardize and validate evaluation. A single physical exam scoring index that correlates with symptomatology and clinical measures should be established, including instructions on systematic scoring to allow intra- and inter-observer consistency and reliability. A standard questionnaire evaluating all aspects of GSM would benefit future research, although this may create a lengthier survey not optimal for clinical use. Developing a short form for the clinical setting would enhance applicability and utility. Methods and cutoff value standardization for pH and VMI would facilitate reproducibility and correlation with other clinical measurements. A standard method of histology specimen collection and assessment could benefit investigating treatment tissue response. Lastly, further elucidation of the genetic pathways altered in menopause is needed to develop targeted therapies.
